# Evolutionary adaptations of a pediatric pathogen: low-inflammatory and high-resistance phenotypes in the emerging *Salmonella typhimurium* monophasic variant 1,4,[5],12:i:-

**DOI:** 10.1128/spectrum.02235-25

**Published:** 2025-09-25

**Authors:** Tengfei Shi, Huahong Qiu, Shaohan Xu, Hui Zhong, Huifang Huang, Huiyu Chen

**Affiliations:** 1 Department of Clinical Laboratory, Fuzhou Second General Hospital 599390 , Fuzhou, Fujian, China; 2 Central Laboratory, Fujian Medical University Union Hospital 117890, Fuzhou, Fujian, China; 3 Department of Clinical Laboratory, Fujian Maternity and Child Health Hospital College of Clinical Medicine for Obstetrics & Gynecology and Pediatrics, Fujian Medical University 74551, Fuzhou, Fujian, China; Children's National Hospital, George Washington University, Washington, DC, USA

**Keywords:** S.1,4,[5],12:i:-, clinical characteristics, molecular genetics, multidrug resistance, whole-genome sequencing, *S. typhimurium*

## Abstract

*Salmonella enterica* serovar 1,4,[5],12:i:- (*S*.1,4,[5],12:i:-), a monophasic variant of *Salmonella typhimurium* (*S. typhimurium*), is an emerging multidrug-resistant pathogen posing a significant threat to pediatric health. Research on this variant remains limited, and due to challenges associated with traditional identification methods, *S*.1,4,[5],12:i:- has often been misclassified as *S. typhimurium*. This study collected clinical data from 122 children infected with *S*.1,4,[5],12:i:- and 42 with traditional *S. typhimurium* in Fujian Province, China, between 2014 and 2023. Whole-genome sequencing was used for strain analysis. Our findings revealed that 77.87% of children with *S*.1,4,[5],12:i:- infection were aged between 1 month and 2 years. Compared with traditional *S. typhimurium*, children with *S*.1,4,[5],12:i:- exhibited milder clinical symptoms, as evidenced by lower levels of the inflammatory marker C-reactive protein (16.53 mg/L *vs*. 33.94 mg/L, *P* < 0.05) and a lower hospitalization rate (26.23% *vs*. 42.86%, *P* < 0.05). These differences may be attributed to the high carriage rate of the anti-inflammatory gene *gogB* in *S*.1,4,[5],12:i:- (95.08% *vs*. 16.67%, *P* < 0.0001). Additionally, *S*.1,4,[5],12:i:- exhibited a higher resistance rate to multiple antibiotics, particularly ceftriaxone, than traditional *S. typhimurium* (32.79% *vs*. 7.14%, *P* < 0.001). This increased resistance may be associated with the carriage of the IncHI2/IncHI2A plasmid. The *S*.1,4,[5],12:i:- ST34 clone prevalent in this region aligns with the global epidemic trend but exhibits greater genetic diversity. Overall, the stealthy evolutionary adaptation of low-inflammation and high-resistance provides novel insights into this variant’s global dominance. These findings underscore the importance of heightened clinical awareness and targeted interventions, particularly for vulnerable pediatric populations.

**IMPORTANCE:**

*S*.1,4,[5],12:i:- poses a growing global health threat, particularly endangering infants and young children. Characterized by increasing prevalence, multidrug resistance, and diagnostic challenges, this variant demonstrates milder inflammatory responses yet stronger antibiotic resistance than traditional *S. typhimurium* in pediatric infections. Crucially, we identified its unique “low-inflammation, high-resistance” evolutionary strategy associated with anti-inflammatory gene *gogB* and resistance plasmid IncHI2/IncHI2A. The stealthy evolutionary adaptation provides novel insights into this variant’s global dominance, while offering critical guidance for improving clinical management and formulating targeted public health measures to protect vulnerable pediatric populations against this cunning pathogen.

## INTRODUCTION

Non-typhoidal *Salmonella* (NTS) is a major cause of bacterial gastroenteritis globally, resulting in hundreds of thousands of infections and deaths each year (1–3). Among the various serovars of NTS, *S. typhimurium* stands out as notable for its tendency to cause enterocolitis in children, characterized by symptoms such as diarrhea and fever (4, 5). In recent years, *S*.1,4,[5],12:i:-, a monophasic variant of *S. typhimurium,* has emerged as a significant global health concern. This variant shares a similar antigenic structure with traditional *S. typhimurium*, which is defined by the serotype formula 1,4,[5],12:i:1,2 and possesses biphasic flagellar antigens (Phase 1: i, Phase 2: 1,2). However, *S*.1,4,[5],12:i:- exhibits only a monophasic flagellar antigen (Phase 1: i) due to genetic mutations that result in the loss of the second-phase flagellar antigen (Phase 2: 1,2). This change has led to its classification as a distinct serotype, typically denoted as *S*.1,4,[5],12:i:- (6–9).

Recent studies have shown that *S*.1,4,[5],12:i:- exhibits enhanced virulence and multidrug resistance (MDR) compared with other NTS serovars, rendering it a more formidable pathogen and increasing the likelihood of antibiotic treatment failure (10–15). Epidemiological data highlight its growing prevalence in pediatric populations: in Greece, it has become the third most common cause of pediatric *Salmonella* infections (16); in Southeast Asia, the ST34 strain of *S*.1,4,[5],12:i:- has emerged as the predominant strain causing both pediatric diarrhea and bloodstream infections (8). In Guangzhou, China, it accounts for 65.24% of pediatric NTS infections (17). Moreover, *S*.1,4,[5],12:i:- has been implicated in numerous outbreaks of foodborne illness, further underscoring its public health significance (15, 18, 19).

Taken together, these characteristics and trends have propelled *S*.1,4,[5],12:i:- to emerge as the predominant serovar causing salmonellosis in humans and animals over the past two decades. This development has presented a significant challenge to both clinical management and public health control. Notably, recent surveillance data from Fujian Province, China, have shown a marked increase in pediatric infections due to *S. typhimurium*. In fact, it now ranks as the most prevalent *Salmonella* serovars (20). However, traditional identification methods have long struggled to rapidly and accurately distinguish *S*.1,4,[5],12:i:- from *S. typhimurium*. As a result, early studies often conflated *S*.1,4,[5],12:i:- with *S. typhimurium*, leading to an underestimation of its true prevalence, clinical severity, and resistance profile. This misclassification has significantly hampered the precision of clinical decision-making and public health interventions.

Children are particularly vulnerable to *Salmonella* infections (16). Although clinicians are increasingly concerned about the epidemiological characteristics of *S*.1,4,[5],12:i:- in pediatric populations, systematic studies on pediatric *S*.1,4,[5],12:i:- infections are still scarce. In particular, the differences in clinical manifestations and antimicrobial resistance between *S*.1,4,[5],12:i:- and typical *S. typhimurium* infections, as well as the molecular genetic mechanisms underlying these differences, have not been fully explored.

This study employed a retrospective analysis of clinical data combined with whole-genome sequencing (WGS) to conduct a comprehensive investigation of isolates from children infected with *S*.1,4,[5],12:i:-. Our primary objectives were to elucidate the epidemiological characteristics, clinical manifestations, antimicrobial resistance profiles, and genomic features of this pathogen. Additionally, we aimed to explore the associations between its phenotypic traits and genomic attributes, thereby providing molecular insights into the interactions between the host and the pathogen. The findings of this study offer a robust scientific basis for optimizing clinical management strategies for pediatric *S*.1,4,[5],12:i:- infections and provide essential data to effectively control its dissemination and inform accurate public health prevention and control measures.

## MATERIALS AND METHODS

### Study design

This study was conducted as a retrospective analysis, focusing on strains of *S*.1,4,[5],12:i:- and *S. typhimurium* isolated from stool samples of pediatric patients at the Maternal and Child Health Hospital of Fujian Province, the largest women’s and children’s specialized hospital in Fujian Province, between January 2014 and December 2023. Cases were carefully selected based on predefined inclusion and exclusion criteria, and comprehensive clinical data were retrieved from medical records. The strains were revived and subjected to antimicrobial susceptibility testing, followed by re-identification using WGS technology. Comparative analyses were performed on key biological indicators, including virulence and resistance genes, and a phylogenetic tree was constructed based on single-nucleotide polymorphisms (SNPs). Ethical approval for this study was obtained from the Ethics Committee of the Maternal and Child Health Hospital of Fujian Province (No. 2024KY248-02).

### Inclusion and exclusion criteria

Inclusion criteria included children aged 14 years or younger who were diagnosed with *S*.1,4,[5],12:i:- or *S. typhimurium* infection, confirmed by stool culture, between January 2014 and December 2023. Only cases with complete clinical records, including detailed medical history, physical examination findings, laboratory test results, and treatment documentation, were included.

Exclusion criteria included children with co-infections involving other pathogens or with underlying medical conditions, such as immunosuppressive states, chronic gastrointestinal diseases, malignant tumors, or other significant comorbidities.

### Collection of clinical data, strain revival, identification, and antimicrobial susceptibility testing

Clinical data for 122 children with *S*.1,4,[5],12:i:- infection and 42 children with *S. typhimurium* infection were extracted from the hospital’s electronic medical record system. The data included demographic characteristics, clinical information, and laboratory indicators. Strains were revived from cryovials stored at −80℃. Re-identification was performed using the Autof MS1000 mass spectrometer (Autobio Diagnostics, Zhengzhou, China) and VITEK-2 gram-negative identification cards (bioMérieux, Inc., Marcy-l’Étoile, France). Preliminary serotyping was performed using slide agglutination combined with a simplified plate method (21), whereas the final serotype confirmation was based on WGS results.

Antimicrobial susceptibility testing was conducted using VITEK-2 antimicrobial susceptibility cards, the disk diffusion method, and the E-test strips to assess susceptibility to 17 antibiotics. The results were interpreted according to the 2024 edition of Clinical and Laboratory Standards Institute guidelines, using *E. coli* ATCC 25922 as the quality control strain.

### Whole-genome sequencing

WGS was performed on the strains by Sangon Biotech (Shanghai, China). Bacterial DNA was extracted using the MagPure Bacterial DNA Kit (Sangon Biotech, Shanghai, China) and quantified with the Qubit dsDNA HS Assay Kit (Thermo Fisher Scientific, Waltham, MA, USA). The DNA was then randomly fragmented into 200–400 bp segments using the Covaris instrument. Libraries were constructed using the Hieff NGS MaxUp II DNA Library Prep Kit (Yeasen Biotechnology, Shanghai, China), purified with magnetic beads, and assessed for concentration and fragment length.

Sequencing was conducted on the Illumina NovaSeq 6000 platform. The sequencing data underwent a series of bioinformatics steps: low-quality reads and adapter sequences were removed using Trimmomatic; quality assessment was performed with FastQC; genome assembly was carried out using SPAdes; gaps were closed with GapFiller; and sequence correction was completed using Pilon. These integrated steps ensured the generation of high-quality genome sequences suitable for downstream analyses.

### Gene annotation and prediction

Serotype prediction was performed using SeqSero 1.2 (https://cge.food.dtu.dk/services/SeqSero/) (22). Multilocus sequence typing (MLST) was conducted using the PubMLST database (https://pubmlst.org/organisms/salmonella-spp), with the species specified as "*Salmonella*" (23). Plasmid analysis was carried out using PlasmidFinder 2.1 (https://cge.food.dtu.dk/services/PlasmidFinder/), with the database specified as "*Enterobacteriales*" (24). Virulence gene annotation was performed using the VFanalyzer tool in the Virulence Factor Database, with the gene species specified as “*Salmonella”* (25). Resistance gene analysis was conducted using ResFinder 4.6 (https://genepi.food.dtu.dk/resfinder), with the species specified as "*Salmonella* spp.*" (26).

### Phylogenetic tree construction

To construct the phylogenetic tree, we selected 21 *S*.1,4,[5],12:i:- strains from three European and American countries, as well as four provinces in China, using EnteroBase (https://enterobase.warwick.ac.uk/). The whole-genome sequences of these strains, along with those of 122 local *S*.1,4,[5],12:i:- strains and 42 *S. typhimurium* strains, were analyzed for SNPs using snp-dists version 0.8.2. The phylogenetic tree was then constructed using the maximum likelihood method implemented in FastTree version 2.1.7 and visualized using ggplot2 version 3.3.2. The tree was further refined and annotated using the online tool tvBOT (https://www.chiplot.online/tvbot.html) (27). Annotations included key information such as sample collection dates, serotypes, stool types (mucus or loose watery stools), C-reactive protein (CRP) levels, and virulence genes.

### Statistical analysis

Statistical analyses were performed using GraphPad Prism version 9.5. For continuous data, normally distributed variables were analyzed using *t*-tests, whereas non-normally distributed variables were assessed using the Mann-Whitney U tests. Categorical data were analyzed using the Chi-square (χ²) tests, with Fisher’s exact test applied when the expected frequency was less than 5. The significance level for all statistical analyses was set at *P* < 0.05.

## RESULTS

### Epidemiological characteristics

**Fig 1:**
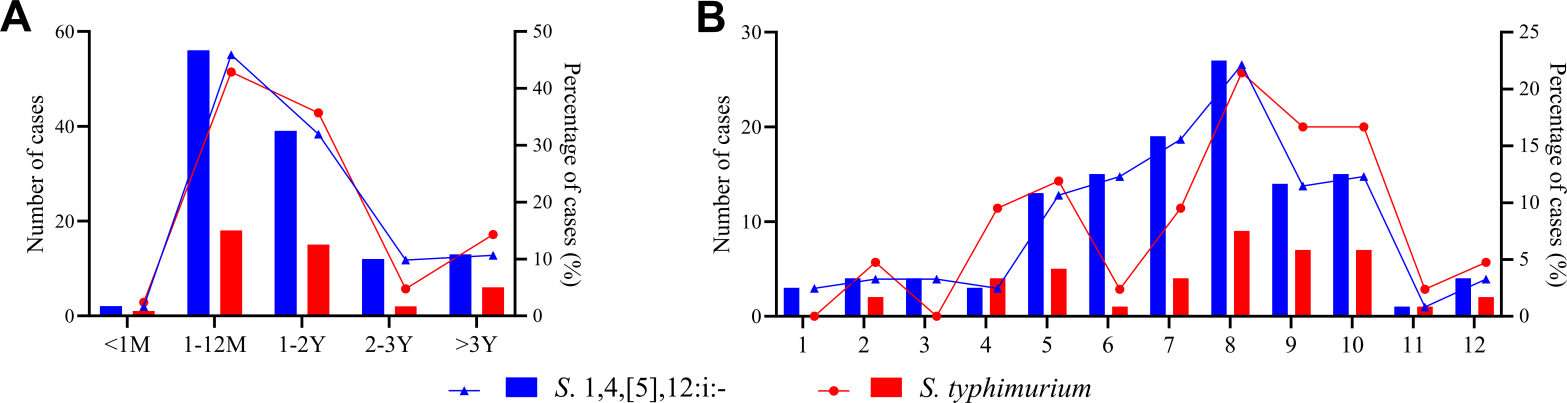
Age and month of infection distribution of 122 children with *S*.1,4,[5],12:i:- and 42 children with *S. typhimurium*. (**A**) Number and percentage of cases by age group. (**B**) Number and percentage of cases by month of infection. Note: The bar chart represents the number of infections, and the line chart represents the proportion (%) of cases in each group.

Children infected with *S*.1,4,[5],12:i:- ranged in age from 4 days to 9 years, with the majority being infants aged 1 month to 2 years (77.87%, 95/122) (Fig. 1A). The male-to-female ratio was 1.44:1, indicating a higher proportion of male patients. The disease exhibited a distinct seasonal pattern, with 84.43% (103/122) of cases occurring between May and October, peaking in August (22.13% of annual cases) (Fig. 1B). Similarly, children infected with *S. typhimurium* had a high incidence among infants aged 1 month to 2 years, with a seasonal peak in the summer and autumn (Fig. 1).

### Clinical manifestations of S.1,4,[5],12:i:- infection

Children infected with *S*.1,4,[5],12:i:- primarily presented with gastrointestinal symptoms (94.3%, 115/122) and fever (81.2%, 99/122), with diarrhea being the most common manifestation. Stools often contained mucus (39.3%, 48/122) or were loose and watery (26.2%, 32/122). Compared with *S. typhimurium*, children with *S*.1,4,[5],12:i:- had significantly lower rates of mucus and loose stools (*P* < 0.05).

Laboratory findings showed that 76.2% (93/122) of children with *S*.1,4,[5],12:i:- had pus cells (≥5/HPF) in their stools, 56.6% (69/122) had red blood cells (≥3/HPF), and 77.9% (95/122) tested positive for occult blood. The median CRP level was 16.5 mg/L, significantly lower than the 33.9 mg/L observed in *S. typhimurium* infections (*P* < 0.05). The median white blood cell count was 10.6 × 10⁹/L (Table 1).

**TABLE 1:** Clinical characteristics of *S*.1,4,[5],12:i:- and *S. typhimurium^a^*

Characteristics	*S.*1,4,[5],12:i:*-* (*n* = 122)	*S. typhimurium* (*n* = 42)	*P*-value
Visit type, n (%)			
Outpatient	90 (73.77)	24 (57.14)	0.04^*^
Inpatient	32 (26.23)	18 (42.86)	0.04^*^
Clinical symptoms, n (%)			
Fever	99 (81.15)	35 (83.33)	0.75
Diarrhea	115 (94.26)	42 (100.00)	0.19
Bloody stool	14 (11.48)	8 (19.05)	0.21
Loose watery stool	32 (26.23)	19 (45.24)	0.02^*^
Respiratory symptoms	23 (18.85)	9 (21.43)	0.72
Vomiting	10 (8.20)	1 (2.38)	0.29
Rash	6 (4.92)	1 (2.38)	0.68
Convulsions	3 (2.46)	1 (2.38)	>0.99
Myocardial injury	2 (1.64)	3 (7.14)	0.27
Hepatic injury	2 (1.64)	1 (2.38)	>0.99
Duration of infection >40 days	7 (5.74)	1 (2.38)	0.68
Blood tests			
WBC, ×10^9^ /L	10.56 (5.49)^*b*^	10.83 (3.52)^*c*^	0.60
NE, %	52.13 (17.13)^*c*^	56.88 (16.56)^*c*^	0.12
CRP, mg/L	16.53 (35.17)^*b*^	33.94 (61.80)^*b*^	0.02^*^
Stool tests, n (%)			
Mucus	48 (39.34)	23 (54.76)	0.04^*^
Pus cells	93( 76.23)	33 (78.57)	0.76
Red blood cells	69 (56.55)	23 (54.76)	0.84
Occult blood	95 (77.87)	34 (80.95)	0.67

^*a*^ WBC, white blood cell count; NE, neutrophil percentage; CRP, C-reactive protein; *P < 0.05.

^*b*^ Median (interquartile range).

^*c*^ Mean (standard deviation).

Although the hospitalization rate was significantly lower for children with *S*.1,4,[5],12:i:- (26.23%) compared with those with *S. typhimurium* (42.86%, *P* < 0.05), chronic diarrhea lasting over 40 days was more frequently observed in *S*.1,4,[5],12:i:- cases (7 cases) than in *S. typhimurium* cases (1 case), with some *S*.1,4,[5],12:i:- cases persisting over 180 days. All cases of chronic diarrhea were confirmed through repeated stool cultures to be positive for the same *Salmonella* serotype. Other intestinal pathogen infections and non-infectious causes (such as inflammatory bowel disease and malabsorption syndromes) had been excluded. The duration of persistent infection was determined based on the date of the last positive *Salmonella* culture.

### High prevalence of the anti-inflammatory gene *gogB*

Children infected with *S*.1,4,[5],12:i:- exhibited milder clinical symptoms compared with those infected with *S. typhimurium*. Further WGS revealed that *S*.1,4,[5],12:i:- harbored 169 virulence factors across 14 categories, with 133 virulence genes detected in all strains (100% prevalence). In contrast, *S. typhimurium* harbored 177 virulence factors across 15 categories, with 141 virulence genes present in all strains. *S*.1,4,[5],12:i:- exhibited fewer types and quantities of virulence genes compared with *S. typhimurium*, with 48 virulence genes differentially distributed between the two serotypes (see Fig. S1).

**Fig 2:**
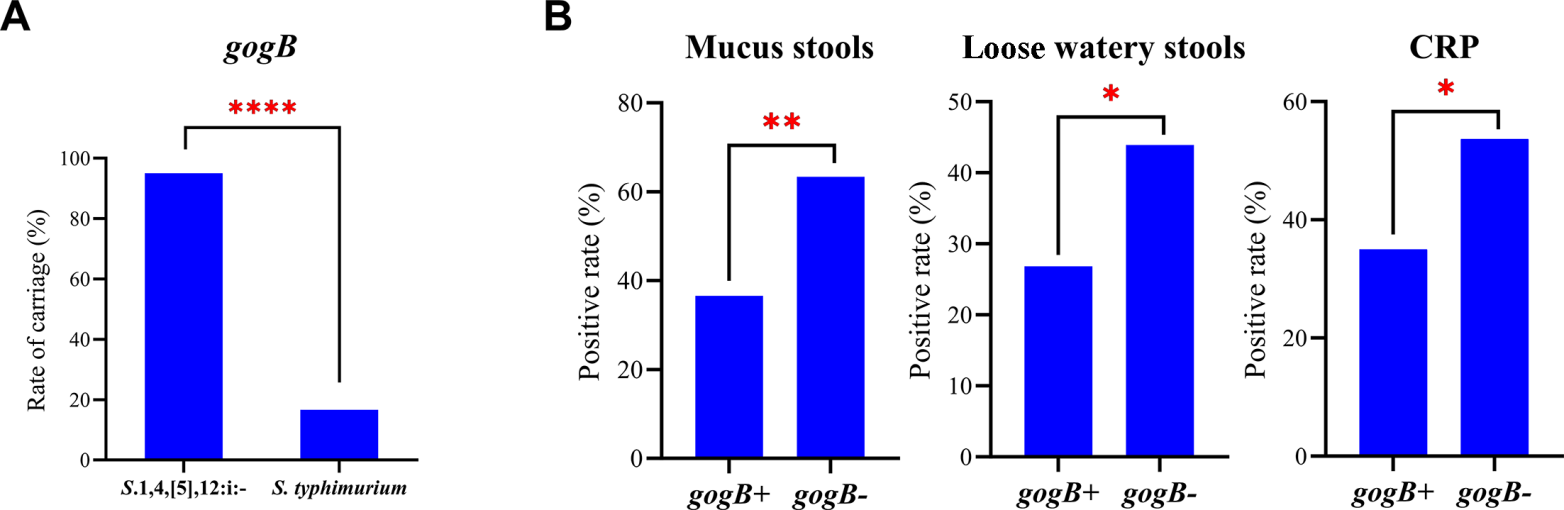
*GogB* is associated with the host inflammatory response. (**A**) Presence of *gogB* gene in *S*.1,4,[5],12:i:- and *S. typhimurium*. (**B**) Host inflammatory response in *gogB*-positive (*gogB*+) and *gogB*-negative (*gogB*-) groups. Note: *: *P* < 0.05；**: *P* < 0.01; ****: *P* < 0.0001.

Notably, the *gogB* gene, known for its anti-inflammatory effects, was detected in 95.08% of *S*.1,4,[5],12:i:- strains, compared with only 16.67% of *S. Typhimurium* strains (*P* < 0.0001) (Fig. 2A). Further analysis showed that the incidence rates of mucus stools, loose watery stools, and CRP levels exceeding 30 mg/L were significantly lower in *gogB*-positive strains than in *gogB*-negative strains (Fig. 2B). These findings suggest that the *gogB* gene may be associated with milder clinical manifestations in children infected with *S*.1,4,[5],12:i:-.

### *S*.1,4,[5],12:i:- exhibits high resistance to multiple antibiotics

In this study, *S*.1,4,[5],12:i:- demonstrated high resistance to multiple antibiotics, with an MDR rate of 89.34% (109/122). The highest resistance rate was observed for ampicillin at 81.15% (99/122), followed by ampicillin-sulbactam at 69.67% (85/122), trimethoprim-sulfamethoxazole at 39.34% (48/122), and ceftriaxone at 32.79% (40/122). Additionally, 6.56% (8/122) of the strains were resistant to azithromycin. In comparison to *S. typhimurium*, *S*.1,4,[5],12:i:- exhibited significantly higher resistance rates to several antibiotics, particularly β-lactam commonly used in pediatric settings, such as ampicillin-sulbactam and ceftriaxone (*P* < 0.05) (Table 2).

**TABLE 2:** Antibiotic resistance for *S*.1,4,[5],12:i:- and *S. typhimurium^a^*

Antibiotic	*S*.1,4,[5],12:i:- (*n* = 122)			*S. typhimurium* (*n* = 42)			*P*-value
	R (%)	I (%)	S (%)	R (%)	I (%)	S (%)	
Ampicillin	81.15	0.00	18.85	78.57	2.38	19.05	0.72
Ampicillin-Sulbactam	69.67	3.28	27.05	47.62	30.95	21.43	0.01^*^
Piperacillin-Tazobactam	0.00	0.82	99.18	2.38	4.76	92.86	0.26
Ceftazidime	12.30	0.82	86.89	7.14	0.00	92.86	0.57
Cefepime	9.02	0.00	90.98	4.76	0.00	95.24	0.52
Ceftriaxone	32.79	0.00	67.21	7.14	0.00	92.86	<0.01^**^
Imipenem	0.00	0.00	100.00	0.00	0.00	100.00	>0.99
Meropenem	0.00	0.00	100.00	0.00	0.00	100.00	>0.99
Levofloxacin	6.56	39.34	54.10	0.00	73.81	26.19	0.12
Ciprofloxacin	11.48	38.52	50.00	16.67	57.14	26.19	0.39
Trimethoprim-Sulfamethoxazole	39.34	0.00	60.66	57.14	0.00	42.86	0.04^*^
Cefoperazone-Sulbactam	5.74	13.11	81.15	2.38	0.00	97.62	0.68
Streptomycin	79.51	9.84	10.66	35.71	42.86	21.43	<0.0001^****^
Nalidixic Acid	25.41	36.89	37.70	16.67	54.76	28.57	0.25
Tetracycline	87.70	0.82	11.48	73.81	2.38	23.81	0.03^*^
Chloramphenicol	49.18	0.82	50.00	69.05	2.38	28.57	0.03^*^
Azithromycin	6.56	0.00	93.44	4.76	0.00	95.24	>0.99

^*a*^ S, susceptible; I, intermediate; R, resistant; *P < 0.05; **P < 0.01; ****P < 0.0001.

Using WGS, we analyzed the resistance genes. *S*.1,4,[5],12:i:- harbored a more complex resistance gene spectrum, with 82 resistance genes across 11 major categories, compared with 60 resistance genes across 10 major categories in *S. typhimurium*. Detailed resistance gene distribution is provided in Table S1 and Fig. S2.

### High ceftriaxone resistance correlates with IncHI2/IncHI2A plasmid carriage

*S*.1,4,[5],12:i:- exhibited significantly higher resistance rates to multiple antibiotics, particularly to ceftriaxone, a core drug for treating pediatric *Salmonella* infections. The ceftriaxone resistance rate was 32.79% in *S*.1,4,[5],12:i:-, compared with 7.14% in *S. typhimurium*, highlighting a concerning divergence in resistance profiles.

**Fig 3:**
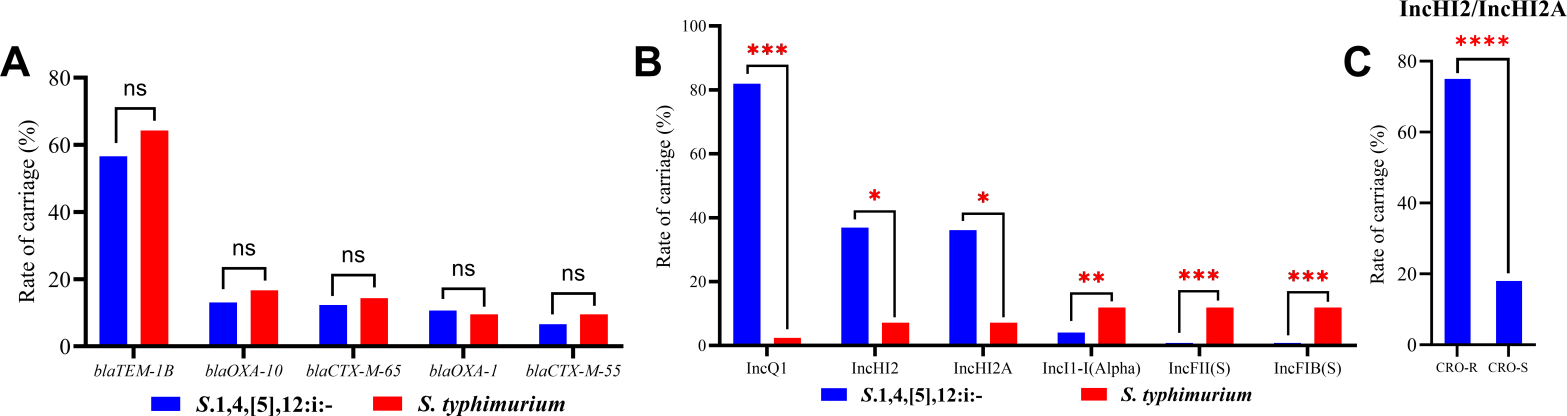
High ceftriaxone resistance is significantly associated with the carriage of the IncHI2/IncHI2A resistance plasmid. (**A**) Comparison of the carriage rates of major β-lactamase resistance genes. (**B**) Distribution differences of major plasmids (IncQ1, IncHI2, IncHI2A in *S*.1,4,[5],12:i:- *vs.* IncFIB(S), IncFII(S), IncI1-I(Alpha) in *S. typhimurium*. (**C**) Carriage rate differences of IncHI2/IncHI2A plasmid between CRO-R and CRO-S groups. Note: *: *P* < 0.05; **: *P* < 0.01; ***: *P* < 0.001; ****: *P* < 0.0001.

To elucidate the reasons for the high ceftriaxone resistance in *S*.1,4,[5],12:i:-, we analyzed the distribution of β-lactamase genes capable of hydrolyzing ceftriaxone. *S*.1,4,[5],12:i:- harbors 28 subtypes of β-lactamase genes across five categories, with *blaTEM-1B* (56.56%) being the most prevalent, followed by *blaOXA-10* (13.11%), *blaCTX-M-65* (12.30%), *blaOXA-1* (10.66%), and *blaCTX-M-55* (6.56%) (Fig. 3A). No significant difference was observed in the proportion of β-lactamase resistance genes between the two serotypes. Beyond resistance genes, we also examined plasmid profiles, given their role as mobile genetic elements that frequently harbor multidrug resistance (MDR) genes and promote resistance dissemination. Strikingly, plasmid distribution differed significantly between *S*.1,4,[5],12:i:- and *S. typhimurium* (*P* < 0.0001). The plasmid carriage rate in *S*.1,4,[5],12:i:- was 95.08% (116/122), which is significantly higher than that in *S. typhimurium*. Notably, the carriage rates of three resistance-associated plasmids—IncQ1 (81.97%, 100/122), IncHI2 (36.89%, 45/122), and IncHI2A (36.07%, 44/122)—were particularly high in *S*.1,4,[5],12:i:- (Fig. 3B).

Further analysis revealed that the carriage rate of IncHI2/IncHI2A plasmids was significantly higher in the ceftriaxone-resistant group (CRO-R) than that in the ceftriaxone-susceptible group (CRO-S) (75.00%, 30/40 vs. 18.29%, 15/82; *P* < 0.0001) (Fig. 3C). This finding indicates a strong association between IncHI2/IncHI2A plasmid carriage and ceftriaxone resistance, suggesting that these plasmids may play a pivotal role in conferring ceftriaxone resistance in *S.*1,4,[5],12:i:-.

### *S*.1,4,[5],12:i:- exhibits high genetic diversity

As a foodborne pathogen with significant regional transmission dynamics, *Salmonella* requires ongoing surveillance of local epidemic clones. In this study, we used MLST and whole-genome SNP-based phylogenetic analysis to identify dominant clones and explore their evolutionary relationships. MLST revealed that the predominant ST34 clone accounted for 96.72% (118/122) *S*.1,4,[5],12:i:- isolates in this region, consistent with the global epidemic trend. In contrast, all *S. typhimurium* strains (42/42) belonged to the ST19 type. Phylogenetic analysis based on core genome SNPs showed that *S*.1,4,[5],12:i:- and *S. typhimurium* formed distinct clades, with *S*.1,4,[5],12:i:- strains distributed across seven clades (C1–C7), reflecting high genetic diversity, whereas *S. typhimurium* strains were clustered in clades C8 and C9.

**Fig 4:**
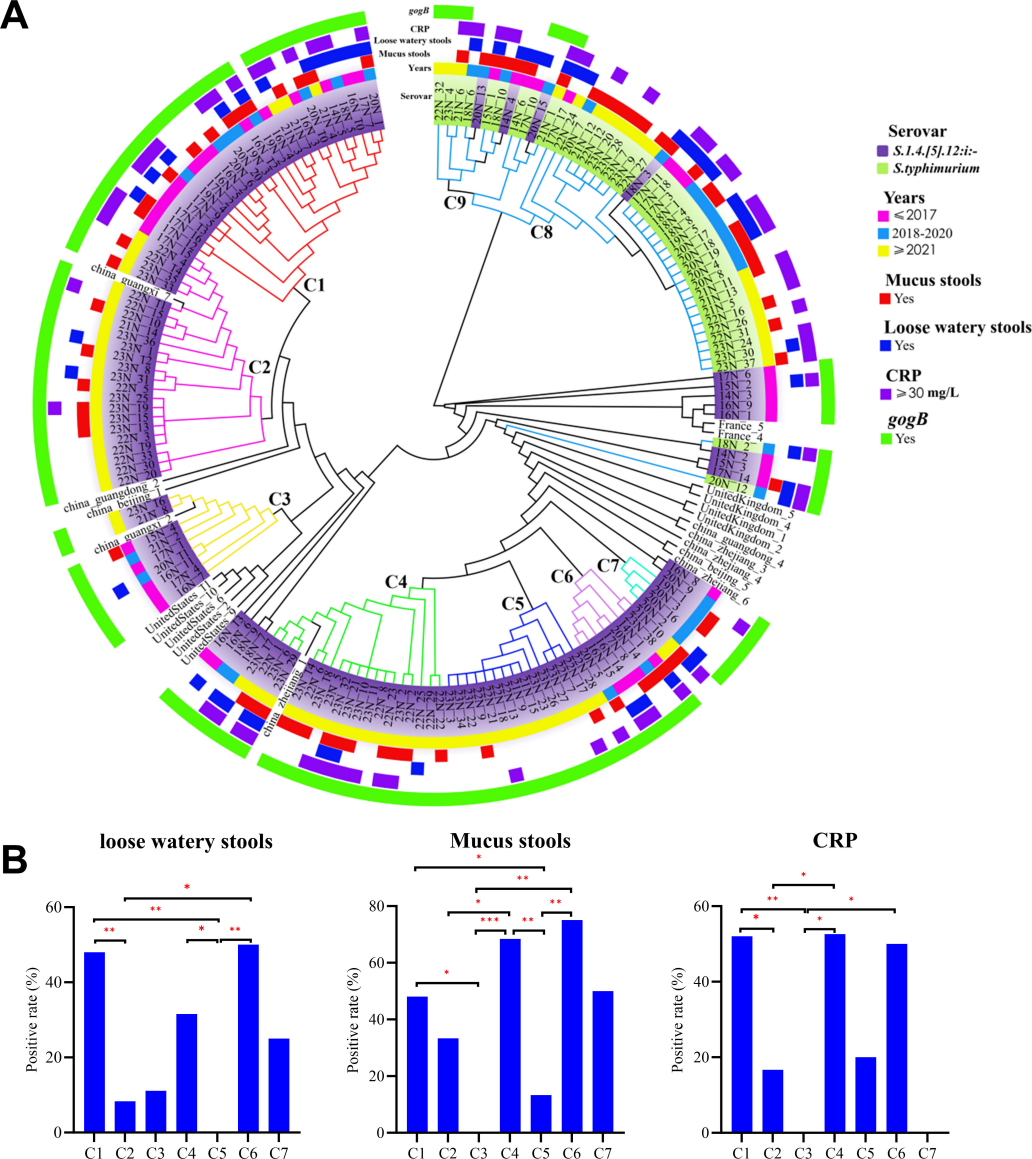
High genetic diversity of *S*.1,4,[5],12:i:-. (**A**) Phylogenetic tree based on SNP analysis showing the genetic relationships and distribution of strains. The circular diagram is organized from the inside out. The innermost ring represents the phylogenetic tree, which is mainly divided into nine clades labeled as C1–C9. Clades C8 and C9 consist of 42 strains of *S. typhimurium*, whereas the remaining 122 strains of *S*.1,4,[5],12:i:- form the clades C1–C7 and some scattered clades. The first ring shows laboratory stock number and serotypes, with the purple color representing *S*.1,4,[5],12:i:-, green for *S. typhimurium*, and uncolored entries indicating strains from other countries and provinces. The second ring indicates the year of strain isolation: pink for before 2017, yellow for after 2021, and blue for the period from 2018 to 2020. The third ring shows the presence of mucus in stool, with red indicating a positive result. The fourth ring shows the presence of loose watery stools, with blue indicating a positive result. The fifth ring indicates CRP levels, with purple representing CRP ≥30 mg/L. The sixth ring shows the distribution of the *gogB* gene, with green indicating detection of the *gogB* gene. (**B**) Significant differences in the positive rates of loose watery stools, mucus stools, and CRP levels among different clades of *S*.1,4,[5],12:i:-. Note: *: *P* < 0.05; **: *P* < 0.01; ***: *P* < 0.001.

Temporal analysis showed that clades C2, C4, and C5 were primarily composed of isolates from 2021 onward, whereas clade C7 was dominated by strains from 2018 to 2020. Clades C1, C3, and C6 contained strains from all years. Notably, clades C2 and C3 formed the same clade with some strains from Guangxi Province, and clade C4 grouped with strains from Zhejiang Province, suggesting regional transmission links (Fig. 4A).

Given the genetic heterogeneity of *S*.1,4,[5],12:i:-, we further examined the association between clades and clinical features. Strains in clades C1, C4, and C6 were more frequently associated with symptoms such as loose watery stools, mucus stools, and elevated CRP levels (Fig. 4B), indicating potential clade-specific differences in virulence or host interaction.

## DISCUSSION

*S*.1,4,[5],12:i:- has progressively replaced conventional *S. typhimurium* and emerged as the predominant epidemic strain, indicating its enhanced competitive fitness and transmission advantages (28–30). The global spread of multidrug-resistant ST34 clones underscores the critical role of antimicrobial resistance in its evolutionary adaptation. However, the clinical impact of this serotype has been underestimated, largely due to limitations in conventional identification methods (31, 32). By integrating retrospective clinical data with WGS, this study provides the first comprehensive characterization of *S*.1,4,[5],12:i:- infections in pediatric patients in Fujian Province, offering valuable insights for clinical management and public health strategies.

Epidemiologically, *S*.1,4,[5],12:i:- infections exhibited distinct age and seasonal patterns. The majority of cases occurred in infants aged 1 month to 2 years, a group with underdeveloped intestinal immune defenses and limited hygiene awareness. The peak incidence during summer and autumn aligns with global trends in foodborne NTS infections (33, 34). As a predominant contaminant in poultry and pork products (18, 35), *S*.1,4,[5],12:i:- poses an increased transmission risk when infants begin consuming complementary foods. Furthermore, the hot and humid climate during these seasons promotes bacterial proliferation (36), reinforcing the link between environmental factors and the epidemiological profile of this serotype.

Clinically, *S*.1,4,[5],12:i:- infections were associated with milder symptoms than those caused by *S. typhimurium*. Affected children exhibited lower incidences of intestinal symptoms, reduced levels of the inflammatory marker CRP, and a significantly lower hospitalization rate. These findings suggest that *S*.1,4,[5],12:i:- elicits a weaker local and systemic inflammatory response, resulting in less severe disease outcomes. This reduced pathogenicity may be linked to the high prevalence of the anti-inflammatory gene *gogB* (37, 38), which aids in modulating host immunity and promotes bacterial survival. Indeed, clinical correlation analysis indicates that children infected with *gogB*-positive strains tended to have milder symptoms and lower inflammatory markers. However, this immune-evading trait may simultaneously contribute to the persistence of infection. Our study found that the proportion of chronic diarrhea caused by *S*.1,4,[5],12:i:- is higher than that caused by *S. typhimurium*. Clinical and molecular analyses of chronic diarrhea cases indicated that its occurrence is not directly driven by specific host factors, evolutionary clades, or genetic backgrounds. Given the significantly higher carriage rate of the *gogB* gene in *S*.1,4,[5],12:i:-, we hypothesize that this serotype may have adopted an evolutionary trade-off strategy: by reducing acute pathogenicity through *gogB*-mediated anti-inflammatory effects, it promotes long-term bacterial colonization and extends the transmission window, thereby achieving an adaptive epidemiological advantage. Although this hypothesis requires further validation, our findings suggest that heightened clinical attention should be paid to the risk of chronic infection with *S*.1,4,[5],12:i:-. Even in children with mild or asymptomatic infections, enhanced monitoring for long-term carriage status and chronic diarrhea is warranted.

The antimicrobial resistance profile of *S*.1,4,[5],12:i:- is particularly concerning. In this study, the ST34 clone accounted for 96.7% of *S*.1,4,[5],12:i:- strains isolated from children in the region, with a MDR rate of 89.34%. This aligns with the global emergence of the MDR ST34 clone (11, 39–41). Resistance is driven by the presence of insertion sequences carrying multiple resistance genes and the carriage of resistance plasmids such as IncQ1, IncHI2, and IncHI2A, which facilitate horizontal gene transfer (8, 12, 42). Of particular concern is the high rate of resistance to ceftriaxone, a first-line antibiotic for pediatric diarrhea (36, 43). Resistance to this agent was significantly higher in *S*.1,4,[5],12:i:- than in *S. typhimurium*, both locally and globally (44, 45). Molecular analysis confirmed that this resistance is closely associated with the IncHI2/IncHI2A plasmid, which commonly harbors extended-spectrum β-lactamase (ESBL) genes, such as *blaCTX-M*, enabling their dissemination among bacterial populations (46, 47). The combined pressure of plasmid-mediated gene spread and clinical overuse of antibiotics has contributed to a sharp rise in ceftriaxone resistance, underscoring the need for targeted surveillance and intervention strategies.

Whole-genome SNP-based phylogenetic analysis further elucidates the potential transmission network of *S*.1,4,[5],12:i:- across China. Local isolates showed close genetic relatedness to strains from other provinces, suggesting that cross-regional transmission is facilitated by food trade and animal product distribution (48, 49). The high genetic diversity of circulating strains, coupled with clade-specific differences in clinical presentation, adds complexity to efforts aimed at controlling the spread of this pathogen.

*S*.1,4,[5],12:i:- has evolved into a highly successful epidemic clone through the combined mechanisms of virulence attenuation and the acquisition of antimicrobial resistance. Its distinctive “low-inflammatory, high-resistance” phenotype likely provides a competitive advantage, facilitating both widespread transmission and host adaptation. These findings offer important insights into the evolutionary strategies that have enabled this pathogen to become a leading cause of pediatric diarrhea globally. Most critically, our results underscore the urgent need for coordinated public health responses. These should include (i) the development of targeted surveillance systems to monitor the transmission dynamics and resistance patterns of this emerging pathogen, and (ii) the implementation of protective strategies for specifically vulnerable infant populations.

This study has several limitations. First, the sample collection was confined to a single medical center, which may affect the generalizability of the findings. Second, although second-generation sequencing can identify resistance genes, it does not provide precise genomic localization, limiting further mechanistic analysis. Third, the absence of information on transmission sources in the clinical data restricts our ability to trace transmission chains. Finally, although the observed ratios reflect the actual epidemiological context, the significant imbalance in sample size between the two serotypes may have influenced the statistical robustness of some comparisons. Future studies should address these limitations to strengthen the validity and broader applicability of the findings.

## Data Availability

The original contributions presented in the study are publicly available. This data can be found here: PRJNA1312295.

## SUPPLEMENTAL MATERIAL

Supplemental material spectrum.02235-25-s0001.pdf Fig. S1 and S2; Table S1.

ASM does not own the copyrights to Supplemental Material that may be linked to, or accessed through, an article. The authors have granted ASM a non-exclusive, world-wide license to publish the Supplemental Material files. Please contact the corresponding author directly for reuse.
