# Supplementary material for: Evolutionary adaptations of a pediatric pathogen: low-inflammatory and high-resistance phenotypes in the emerging Salmonella typhimurium monophasic variant 1,4,[5],12:i:-
Source: Microbiol Spectr. 2025 Sep 25;13(11):e02235-25. doi: 10.1128/spectrum.02235-25 (PMC12584699; doi:10.1128/spectrum.02235-25)
Supplement: Supplemental material — Fig. S1 and S2; Table S1. [file spectrum.02235-25-s0001.pdf]

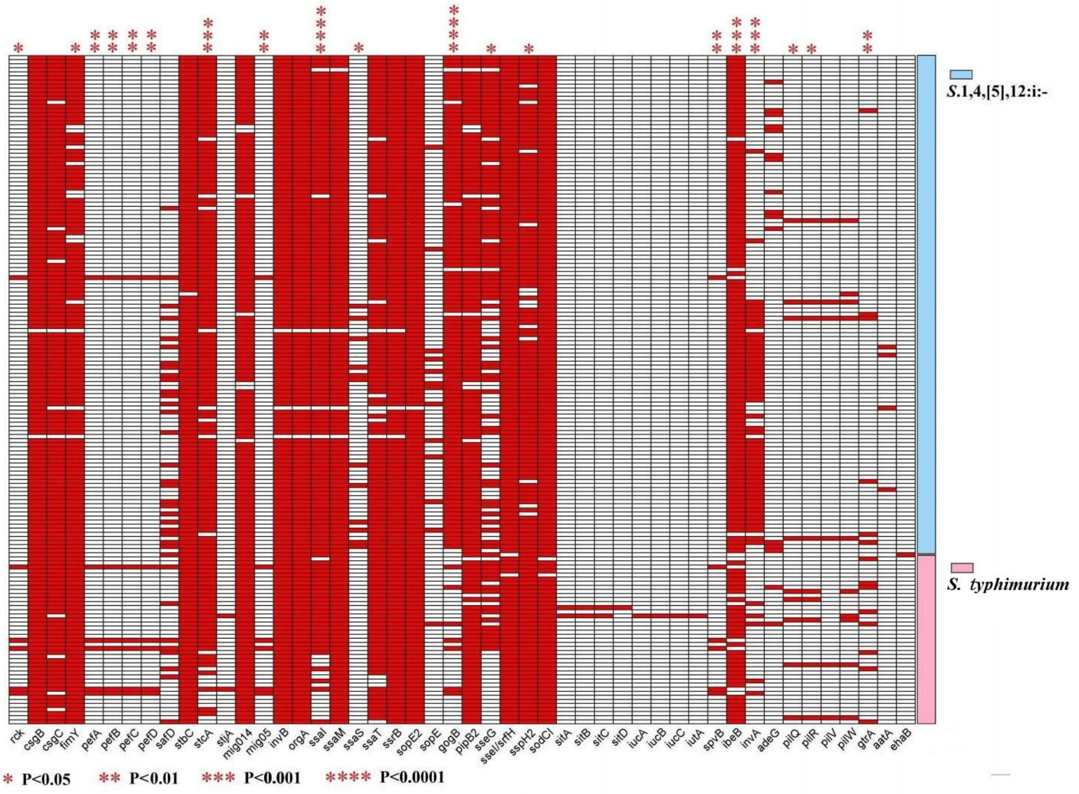


FIG S1 Heatmap of differentially harbored virulenc genes between *S.*1,4,[5],12:i:- and *S. typhimurium.* This heatmap illustrates the distribution of 48 virulence genes between *S.*1,4,[5],12:i:- (blue sidebar) and *S. typhimurium* (pink sidebar), with rows representing individual strains and columns representing virulence genes (labeled below). Red cells denote gene presence, while white indicates absence. The *gogB* gene prevalence differed significantly: 95.08% in *S.*1,4,[5],12:i:- vs. 16.67% in *S. typhimurium* ($p < 0.0001$) .

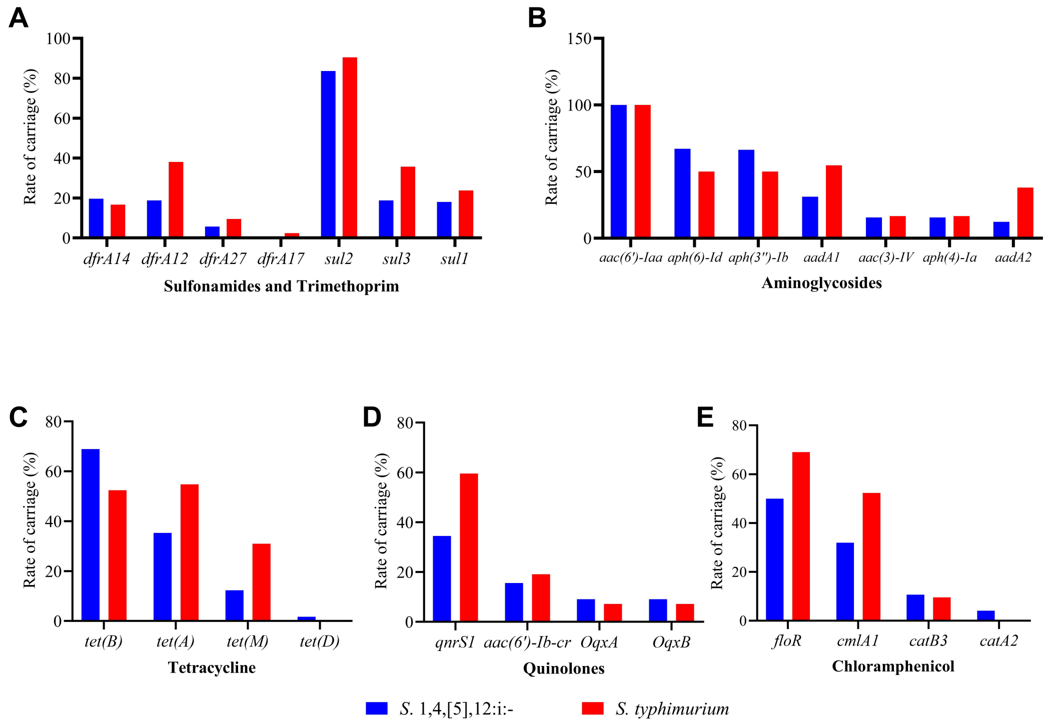


**FIG S2** Prevalence of major antibiotic resistance genes against different antibiotic classes. (A) Comparison of the carriage rates of major Sulfonamides and Trimethoprim resistance genes. (B) Comparison of the carriage rates of major Aminoglycosides resistance genes. (C) Comparison of the carriage rates of major Tetracycline resistance genes. (D) Comparison of the carriage rates of major Quinolones resistance genes. (E) Comparison of the carriage rates of major Chloramphenicol resistance genes.

**TABLE S1** Summary of the carriage rates of major antibiotic resistance genes in *S.*1,4,[5],12:i:- and *S. typhimurium*

| Antibiotic Class | Resistance Genes | *S.*1,4,[5],12:i:- (n=122), n(%) | *S. typhimurium* (n=42), n(%) | *p*-value |
|---|---|---|---|---|
| **β-Lactams** | *blaCMY-2, blaCTX-M-14, blaCTX-M-55, blaCTX-M-65, blaDHA-1*[#]*, blaOXA-1, blaOXA-10, blaTEM-104, blaTEM-126*[#]*, blaTEM-128*[#]*, blaTEM-141*[#]*, blaTEM-148, blaTEM-176, blaTEM-186*[#]*, blaTEM-198, blaTEM-1A*[#]*, blaTEM-1B, blaTEM-30, blaTEM-70, blaTEM-206*[#]*, blaTEM-207, blaTEM-209*[#]*, blaTEM-214*[#]*, blaTEM-216*[#]*, blaTEM-217, blaTEM-220*[#]*, blaTEM-230, blaTEM-234* | 99(81.15) | 36(85.71) | 0.50 |
| **Aminoglycosides** | *aac(2')-IIa*[#]*, aac(3)-IIa*[Δ]*, aac(3)-IId, aac(3)-IV, aac(6')-Iaa, aadA1, aadA16, aadA17*[#]*, aadA2, aadA22, aadA24, aadA2b, aadA5, aadA8b, ant(2'')-Ia*[Δ]*, ant(3'')-Ia, aph(3')-Ia, aph(3'')-Ib, aph(4)-Ia, aph(6)-Id* | 122(100.00) | 42(100.00) | >0.99 |
| **Macrolides** | *erm(B)*[#]*, mph(A)* | 7(5.74) | 1(2.38) | 0.68 |
| **Polymyxins** | *mcr-1*[#] | 3(2.46) | 0(0.00) | 0.57 |
| **Quinolones** | *OqxA, OqxB, qnrA1, qnrB4*[#]*, qnrB6*[Δ]*, qnrS1, qnrS10*[#]*, qnrS2*[#]*, qnrS3*[#]*, aac(6')-Ib-cr* | 58(47.54) | 33(78.57) | < 0.01[**] |
| **Rifamycins** | *ARR-2, ARR-3, ARR-6*[#]*, bleO* | 44(36.07) | 17(40.48) | 0.61 |
| **Lincosamides** | *lnu(F)* | 9(7.38) | 3(7.14) | >0.99 |
| **Fosfomycins** | *fosA3* | 4(3.28) | 1(2.38) | >0.99 |
| **Phenicols** | *catA2*[#]*, catB3, cmlA1, floR* | 65(53.28) | 30(71.43) | 0.04[*] |
| **Tetracyclines** | *tet(A), tet(B), tet(D)*[#]*, tet(M)* | 110(90.16) | 39(92.86) | 0.76 |
| **Sulfonamides** | *dfrA12, dfrA14, dfrA17, dfrA27, sul1, sul2, sul3* | 107(87.7) | 39(92.86) | 0.56 |

Notes: [#]: Exclusive to *S.*1,4,[5],12:i:-; [Δ]: Exclusive to *S. typhimurium*; *: $p < 0.05$; **: $p < 0.01$
